# The p.Arg52Leu variant in *GNB2* causes a gain-of-function due to an increase of I_K, ACh_ in stimulated hiPSC-CMs

**DOI:** 10.1186/s12872-026-06735-3

**Published:** 2026-10-05

**Authors:** Arie O. Verkerk, Anne Kayser, Jassin Hamidi, Sven Dittmann, Eric Schulze-Bahr

**Affiliations:** 1 Department of Medical Biology, Amsterdam University Medical Centers, University of Amsterdam, Amsterdam, The Netherlands; 2 Department of Experimental Cardiology, Amsterdam University Medical Centers, University of Amsterdam, Amsterdam, The Netherlands; 3 Institute for Genetics of Heart Diseases (IfGH), University Hospital Münster, Münster, Germany

**Keywords:** Sinus node dysfunction, *GNB2*, GIRK, Patch clamp, Spontaneous activity, Blocker, hiPSC

## Abstract

**Background:**

Initially discovered and published in 2017, the heterozygous mutation (p.Arg52Leu) in the *GNB2* gene is associated with sinus node dysfunction (SND) and atrioventricular block (AVB). Electrophysiological characterization using heterologous expression systems has demonstrated that this R52L *GNB2* variant results in a larger G-protein–activated K^+^ (GIRK/I_K, ACh_) current. So far, this has not been functionally validated in patient-derived disease modelling using human induced pluripotent stem cell-derived cardiomyocytes (CM).

**Methods:**

Here, we differentiated CMs derived from human induced pluripotent stem cells (hiPSC-CMs) of a patient carrying the pathogenic p.Arg52Leu *GNB2* variant (hiPSC^R52L^-CMs) and compared electrophysiological properties with those of two unrelated healthy hiPSC-CMs control lines. Retinoic acid (RA) treatment during the differentiation was used to generate hiPSC-CMs with robust acetylcholine-activated K^+^ currents (I_K, ACh_).

**Results:**

Patch-clamp experiments demonstrated that without GIRK channel stimulation the maximal diastolic potential (MDP) and spontaneous pacing rate did not differ between mutant and control cell lines and, moreover, a constitutively active I_K, ACh_ was not observed. Upon stimulation of GIRK channels with carbachol (CCh), hiPSC^R52L^-CMs displayed an increased I_K, ACh_ density, a more hyperpolarized MDP and a slower spontaneous activity as compared to both control hiPSC-CMs cell lines, thus explaining the bradycardia reported from patients carrying the R52L *GNB2* variant. Application of the specific I_K, ACh_ blocker XAF-1407 rescued the phenotype.

**Conclusion:**

The reported, mutant *GNB2* in patient-derived CMs is associated with a gain-of function upon GIRK channel stimulation and thereby a larger I_K, ACh_, which could be potentially targeted and rescued by I_K, ACh_ blockers.

**Clinical trial number:**

not applicable.

## Introduction

The acetylcholine-activated K^+^ current (I_K, ACh_) is a key player in heart rate modulation via parasympathetic signaling [1]. I_K, ACh_ in the heart is conducted through heterotetramers of two G-protein activated inwardly rectifying K^+^ channels, GIRK1 (also named Kir3.1) and GIRK4 (also named Kir3.4), whose α-subunits are encoded by *KCNJ3* and *KCNJ5* genes, respectively [2]. These channels are abundant in the sinoatrial node (SAN) and atria [3] and virtually absent in the ventricle [1, 4, 5]. Activation of I_K, ACh_ via muscarinic M2-receptor binding induces an outwardly directed K^+^ current [6, 7] which in SAN cells hyperpolarizes the maximal diastolic potential (MDP) and reduces the speed of the diastolic depolarization phase, resulting in an increased intrinsic SAN cycle length [8, 9] and thus heart rate reduction. I_K, ACh_ activation in atrial cardiomyocytes (CMs) also hyperpolarizes the resting membrane potential (RMP) [10], although to a smaller content than in SAN cells [11], because the RMP in atrial is more close to the reversal potential of K^+^ (E_K_) currents [12]. Nevertheless, the atrial RMP hyperpolarization may also affect heart rate generation, since it reduces the excitability of atrial cells [13].

It is increasingly recognized that mutations in genes encoding I_K, ACh_ channel subunits or genes which modulate its function, may result in inherited arrhythmia disorders [13–16], in particular sinus node dysfunction (SND). SND, previously known as sick sinus syndrome (SSS), is clinically manifested in sinus bradycardia, sinus pause, and sinus arrest as a cause of impaired SAN impulse generation, and/or SAN exit block due to delayed or absent impulse conduction between the SAN and atria [17–19]. So far, three I_K, ACh_-related gene variants are linked to SND. First, the *KCNJ5* variant W101C has been reported as a gain-of-function variant in a family with SND [15]. Functional data in heterologous systems (CHO cells and Xenopus oocytes), and human induced pluripotent stem cell derived-cardiomyocytes (hiPSC-CMs) demonstrated a constitutively active I_K, ACh_ under baseline conditions [15, 16], resulting in a more negative MDP and a slower spontaneous pacing frequency. The other gene variants linked to I_K, ACh_-related SND are in guanine nucleotide-binding proteins (*GNBs*) [13, 14], from which it is known that they modulate I_K, ACh_ function importantly (for review, see [7, 20, 21]. The *GNB* subunit beta-5 (*GNB5*) variant, S81L, did not affected the electrophysiological properties without GIRK stimulation, but resulted in a larger I_K, ACh_ in hiPSC-CMs upon GIRK stimulation, leading to a large MDP hyperpolarization and cessation of spontaneous pacemaking [13]. The *GNB* subunit beta-*2* (*GNB2*) variant, R52L, is also a gain-of-function mutation based on functional data obtained in heterologous expression systems, e.g., CHO cells and Xenopus oocytes [14]. Stallmeyer and colleagues [2017] found larger GIRK currents when they expressed Kir3.1/Kir3.4 in the presence of Gγ2, with the R52L *GNB2* variant [15]. It is unknown if this can be considered as constitutively active I_K, ACh_, since the expression data with Kir3.1/Kir3.4, Gγ2, and wild-type GNB2 already resulted in a basal current without muscarinic receptor stimulation which contrasts findings in CMs.

In the present study, we characterized further the potential relevance of the R52L *GNB2* variant in a patient-derived cardiomyocyte environment. Therefore, we examined hiPSC-CMs of a patient carrying this *GNB2* variant and suffering from SND, which were previously published and named hiPSC^R52L^-CMs hereafter [22]. Typically, hiPSC-CMs have a depolarized MDP and are spontaneously active, largely similar as human sinoatrial node cells [23, 24], and have been used as ‘pacemaker’ cell model before [13, 16, 25]. Retinoic acid (RA) application during the differentiation process was used to generate hiPSC-CMs with robust I_K, ACh_ upon GIRK stimulation [13, 16, 26–31], making them useful for studying GIRK channelopathies. We found that in absence of GIRK stimulation, the AP properties of hiPSC^R52L^-CMs were largely similar to two unrelated healthy control (CTRL) hiPSC-CMs lines. In addition, baseline I_K, ACh_ activity was neither observed in the CTRL hiPSC-CMs nor in hiPSC^R52L^-CMs. However, upon GIRK stimulation with 10 µM carbachol (CCh), we found a larger I_K, ACh_ density in hiPSC^R52L^-CMs as compared to the CTRL hiPSC-CMs. This resulted in more pronounced effects of CCh on APs, especially on MDP and spontaneous pacing frequency. Blockade of I_K, ACh_ using the highly selective GIRK channel blocker XAF-1407 supressed the gain-of-function of the hiPSC^R52L^-CMs underlining its use as a potential treatment option for patients suffering from severe bradycardia caused by GIRK channel gain-of-function mutations.

## Materials and methods

### Cell preparation

hiPSCs from a patient with the R52L *GNB2* variant were generated and characterized previously in detail [22]. This line is registered in the Human Pluripotent Stem Cell Registry (hPSCreg^®^ number: UKMi003-A). From this patient line, we generated two isogenic control hiPSC lines with CRISPR/Cas9-mediated genome editing [22], but unfortunately one clone (K6) has defects in chromosome 1 (data not shown), and the other clone (K26) was virtually impossible to differentiate towards CMs with our standard differentiation protocol. Therefore, two unrelated healthy hiPSC lines (CTRL1 and CTRL2) that were generated for previous studies in which we participated by carrying out patch-clamp experiments [13, 16, 32, 33] served as control. These lines are also registered in the hPSCreg^®^ (CTRL1: UKMi006-A; CTRL2: UMGi055-A.4). To induce I_K, ACh_ expression in hiPSC-CMs, 0.5-1.0 µM all-trans-retinoic acid (RA; Sigma) was applied during the differentiation [13, 16, 32]. DMSO-treated hiPSC-CMs served as CMs without I_K, ACh_ expression. Dissociation of CTRL1- and hiPSC^R52L^-CMs was similar as described previously by Kayser et al. [16]; CTRL2-hiPSC-CMs dissociation was as described by Veerman et al. [13].

### Patch clamp experiments

#### Data acquisition

Electrophysiological measurements were performed on single hiPSC-CMs that showed spontaneous and regular beating upon visual inspection, clearly indicating a healthy and myocardial status [32]. Action potentials (APs) and membrane currents were recorded at 36 ± 0.2 °C with the amphotericin-perforated patch-clamp technique using an Axopatch 200B amplifier (Molecular Devices, Sunnyvale, CA, USA). Signals were low-pass filtered with a cut-off of 5 kHz, and digitized at 5 kHz. Voltage control, data acquisition, and analysis were realized with custom software. The potentials were corrected for the calculated liquid junction potential [34]. Cells were superfused with modified Tyrode’s solution containing (in mM): NaCl 140, KCl 5.4, CaCl_2_ 1.8, MgCl_2_ 1.0, glucose 5.5, and HEPES 5.0; pH was set to 7.4 with NaOH. Patch pipettes were pulled from borosilicate glass (Harvard apparatus, UK) and had tip resistances of 2.0–3.0 MΩ after filling with the pipette solution containing (in mM): K-gluc 125, KCl 20, NaCl 5, amphotericin-B 0.44, and HEPES 10; pH was set to 7.2 with KOH. Cell membrane capacitance (C_m_) was estimated by dividing the time constant of the decay of the capacitive transient in response to 5 mV hyperpolarizing voltage clamp steps from − 40 mV by the series resistance. For membrane current measurements, series resistance was compensated by ≥ 70%. Detailed current and voltage clamp measurements were performed when the recordings were stable.

#### Current clamp experiments

APs were recorded under baseline conditions and after application of 10 µM CCh to induce fully GIRK stimulation [13, 16, 26]. APs were characterized by spontaneous pacing frequency, MDP, maximum upstroke velocity (V_max_), maximum AP amplitude (APA), AP plateau amplitude (APA_plat_ - defined as the potential difference between MDP and potential at 20 ms after the V_max_), and the duration at 20, 50, and 90% repolarization (APD_20_, APD_50_, and APD_90_, respectively). Parameter values obtained from 10 consecutive APs were averaged.

#### Voltage clamp experiments

Membrane currents were measured during a descending RAMP protocol from 20 to -120 mV from a holding potential of -40 mV [16, 26]. We measured membrane currents under baseline conditions and in the presence of 10 µM CCh. CCh-sensitive currents were obtained by subtracting the currents recorded in presence of CCh from those recorded under baseline conditions. Current densities were calculated at fixed potential, i.e., -120 mV, -110 mV, etc. (Fig. 3) by dividing current amplitudes by C_m_.

### Statistics

Data are presented as mean ± SEM. Statistical analysis was carried out with SigmaStat 3.5 software (Systat Software, Inc., San Jose, CA, USA). Two groups were compared with a paired (Wilcoxon Signed Rank Test) or an unpaired (Mann–Whitney Rank Sum Test) rank test, and more than 2 groups with the Kruskal-Wallis test followed by Dunn’s post-hoc test or by the Two-way repeated measures (RM) ANOVA followed by Bonferroni post-hoc test. *P* < 0.05 was considered as statistically significant.

## Results

### hiPSC^R52L^-CMs lack a sustained I_K, ACh_ activation under baseline conditions

**Fig. 1:**
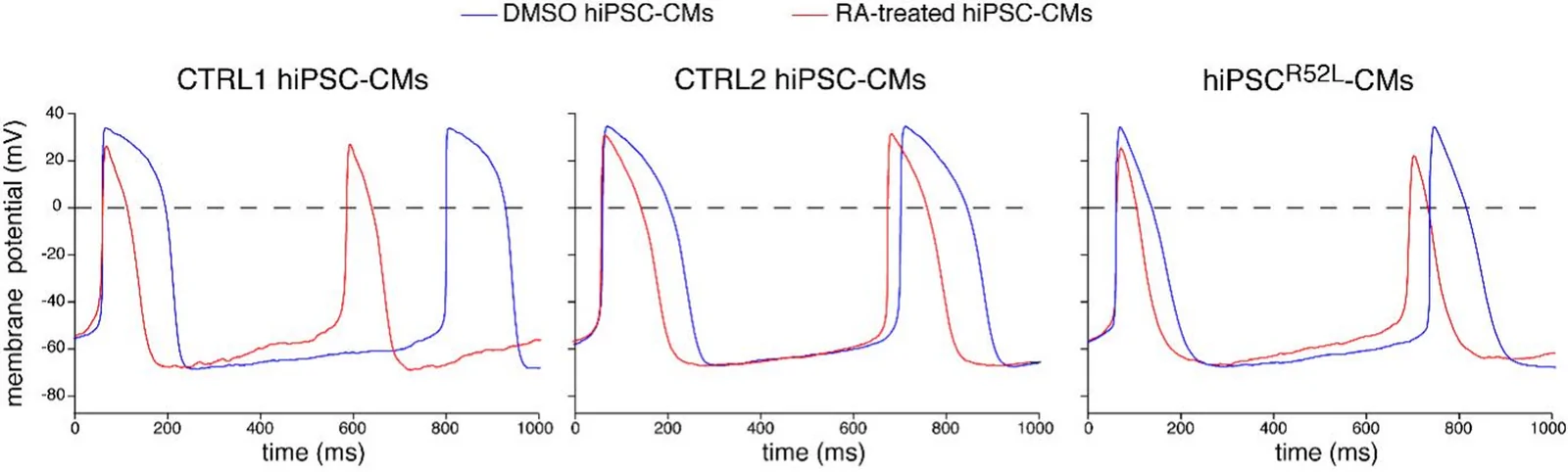
Typical action potential traces in DMSO- and RA-treated CTRL1 hiPSC-CMs, CTRL2 hiPSC-CMs, and hiPSC-CMs carrying the R52L *GNB2* variant (hiPSC^R52L^-CMs)

In the present study, we performed patch clamp measurements on single hiPSC-CMs that showed spontaneous and regular AP formation with a clear diastolic depolarization phase (Fig. 1), largely similar as isolated human sinoatrial node cells [23, 27]. hiPSC-CMs were generated with standard differentiation protocols (with DMSO as vehicle) and in the presence of RA during the differentiation to induce robust I_K, ACh_ expression [13, 22, 26–31], which was also valid for the current study (see below, Sect. GIRK stimulation results in larger I_K, ACh_ in hiPSC^R52L^-CMs). The RA differentiation resulted in all hiPSC-CMs lines in APs with a faster repolarization (Fig. 1).

Consequently, the RA-treated hiPSC-CMs have a decreased average APD_20_ and APD_50_ and a decreased average AP plateau amplitude as compared to the standard (DMSO) differentiation protocol (Table 1). This is in agreement with many previous studies in which RA was used during the differentiation protocol (for overview, see ref [35]; and primary references cited therein). The AP parameters of the DMSO-treated CMs did not differ between the three lines (Table 1; *P* > 0.05 (Kruskal-Wallis test followed by Dunn’s test)). In the RA-treated CMs, CTRL2 hiPSC-CMs had a higher AP plateau amplitude than hiPSC^R52L^-CMs as well as increased APD_20_, APD_50_ compared to CTRL1- and hiPSC^R52L^-CMs (Table 1), likely due to some basic background differences between the lines.

**Table 1:** Baseline action potential (AP) parameters of RA- and DMSO-treated hiPSC-CMs

	CTRL1 hiPSC -CMs		CTRL2 hiPSC-CMs		hiPSC^R52L^-CMs	
	DMSO (*n* = 10)	RA (*n* = 11)	DMSO (*n* = 15)	RA (*n* = 19)	DMSO (*n* = 13)	RA (*n* = 14)
MDP (mV)	-70.7 ±0.6	-69.3 ±0.7	-68.2 ±1.3	-68.7 ±1.0	-68.0 ±1.1	-68.3 ±1.2
APA (mV)	95.1 ±4.2	91.8 ±2.5	103.4 ±3.1	93.6 ±3.2	97.5 ±3.4	83.1 ±3.7*
AP Plat (mV)	94.6 ±3.6	71.5 ±5.8*	98.7 ±3.3	86.5 ±3.3*	93.8 ±3.5	53.8 ±8.1*^,#^
V_max_ (V/s)	62.5 ±15.0	55.6 ±16.2	60.0 ±18.9	32.3 ±7.5	44.9 ±11.9	34.8 ±6.4
APD_20_ (ms)	79.1 ±9.1	27.0 ±6.2*^,#^	88.4 ±11.3	52.2 ±3.9*	86.8 ±12.2	30.6 ±9.6*^,#^
APD_50_ (ms)	122.4 ±14.1	53.7 ±10.9*^,#^	146.7 ±18.4	91.1 ±6.2*	134.7 ±17.7	53.7 ±15.0*^,#^
APD_90_ (ms)	140.0 ±14.8	112.4 ±19.2^=^	186.1 ±18.5	135.6 ±8.8*	166.5 ±19.8	166.2 ±114.7
Freq (Hz)	1.2 ±0.1	2.0 ±0.2*	1.6 ±0.2	2.0 ±0.1*	1.7 ±0.2	1.7 ±0.1

Average ±SEM. MDP = maximal diastolic potential; AP = action potential; APA = AP amplitude; AP plat = plateau amplitude measured at 20 ms after V_max_; V_max_ = maximal AP upstroke velocity; APD_20_, APD_50_, APD_90_ = AP duration at 20, 50, and 90% of repolarization; Freq = frequency. * *P* < 0.05 DMSO vs. RA (Mann Whitney test); ^#^ *P* < 0.05 vs. RA CTRL2-CMs (Kruskal-Wallis followed by Dunn’s test); ^=^ *P* < 0.05 vs. RA hiPSC^R52L^-CMs (Kruskal-Wallis followed by Dunn’s test)

**Fig. 2:**
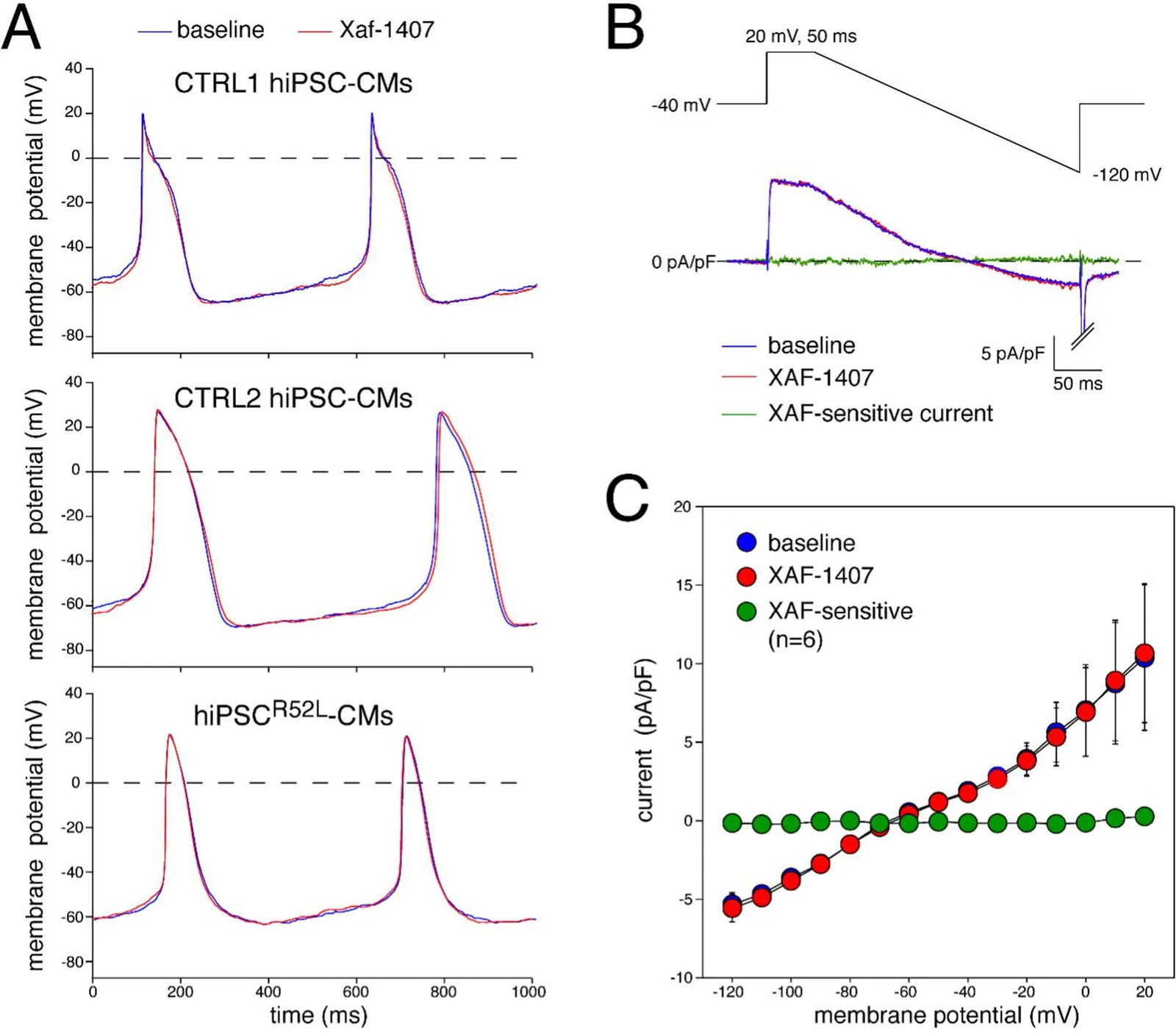
Effect of XAF-1407 on RA-treated hiPSC-CMs.** A** Typical APs of all three hiPSC-CMs lines in absence (baseline) and presence of 100 nM XAF-1407. **B** Typical current traces recorded in a RA-treated hiPSC^R52L^-CM during a RAMP protocol (top panel) in absence (baseline) and presence of XAF-1407 as well as the XAF-sensitive current. **C** Average current-voltage (I-V) relationships in absence, in presence of XAF-1407, and the XAF-sensitive current

Relevant for SND-related GIRK channelopathies, the MDP and the spontaneous pacing frequency of the RA-treated CMs hiPSC^R52L^-CMs was similar to those of the RA-treated CTRL lines (*P* > 0.05; Kruskal-Wallis test) (Table 1), which is in contrast to CMs with constitutively active I_K, ACh_ in absence of GIRK stimulation, such as RA-treated hiPSC-CMs with a *KCNJ5* mutation [16]. This suggests that the R52L *GNB2* mutation does not result in a persistent I_K, ACh_ current under baseline conditions. To examine this in more detail, we next measured the effects of the selective I_K, ACh_ channel blocker, XAF-1407 [16, 36–38], on AP properties of RA-treated hiPSC-CMs. Typical examples are shown in Fig. 2A. 100 nM XAF-140, a concentration which fully blocked KIr currents without affecting other membrane currents 36, neither affected the AP properties of the CTRL1-, and CTRL2-hiPSC-CMs nor those of hiPSC^R52L^-CMs (Table 2; *P* > 0.05 (Wilcoxon signed rank test)). Especially, the lack of XAF-1407 effects on MDP and pacing frequencies indicated that I_K, ACh_ was not constitutively active in our RA-treated hiPSC-CMs. This is further substantiated in voltage clamp experiments where we used a descending ramp protocol (Fig. 2B, top panel) and tested for XAF-sensitive currents in RA-treated hiPSC^R52L^-CMs, similarly as has been shown for the *KCNJ5* mutation in hiPSC-CMs [16]. The recorded currents in the absence (baseline) and in presence of XAF-1407 were virtually overlapping (Fig. 2B, bottom panel). Consequently, XAF-sensitive currents were absent. Figure 2C shows average current-voltage (I-V) relationships of six cells under baseline and in the presence of XAF-1407, and these did not differ significantly (*P* > 0.05; Two-way RM ANOVA). Thus, average XAF-sensitive currents were around the zero-current level (Fig. 2C). In summary, these data demonstrate that without GIRK stimulation a constitutively active I_K, ACh_ is absent in cardiomyocytes with the R52L *GNB2* variant.

**Table 2:** Effect of 100 nM XAF-1407 on AP parameters of RA-treated CTRL1 hiPSC-CMs, CTRL2 hiPSC-CMs, and hiPSC^R52L^-CMs

	CTRL1 hiPSC –CMs (*n* = 3)		CTRL2 hiPSC-CMs (*n* = 5)		hiPSC^R52L^-CMs (*n* = 4)	
	baseline	XAF-1407	baseline	XAF-1407	baseline	XAF-1407
MDP (mV)	-70.5 ±1.4	-70.4 ±1.2	-69.2 ±2.7	-69.7 ±2.8	-66.1 ±2.2	-66.6 ±1.6
APA (mV)	86.6 ±8.7	86.6 ±8.9	88.7 ±7.7	89.1 ±8.1	82.8 ±7.8	82.1 ±7.6
AP Plat (mV)	76.2 ±4.1	72.5 ±2.6	82.3 ±9.4	83.6 ±9.5	64.3 ±7.7	65.4 ±6.9
V_max_ (V/s)	45.5 ±43.9	54.7± 54.8	7.6 ±2.2	7.8 ±2.7	46.7±17.2	46.5 ±17.5
APD_20_ (ms)	30.7 ±8.4	23.8 ±7.2	61.6±5.9	63.1 ±7.0	22.4± 8.7	22.3 ±8.1
APD_50_ (ms)	68.6 ±22.1	62.7 ±24.8	101.7± 10.5	104.9 ±11.3	41.2 ±10.6	41.6 ±8.9
APD_90_ (ms)	104.6 ±33.4	102.7 ±31.3	141.7 ±15.3	147.3 ±17.0	135.6 ±18.2	126.5 ±16.9
Freq (Hz)	1.7 ± 0.3	1.7 ±0.3	2.2 ±0.3	2.1 ±0.3	1.8± 0.1	1.8 ±0.2

Average ±SEM. MDP = maximal diastolic potential; AP = action potential; APA = AP amplitude; AP plat = plateau amplitude measured at + 20 ms after V_max_; V_max_ = maximal AP upstroke velocity; APD_20_, APD_50_, APD_90_ = AP duration at 20%, 50%, and 90% of repolarization; Freq = frequency

### GIRK stimulation results in larger I_K, ACh_ in hiPSC^R52L^-CMs

**Fig. 3:**
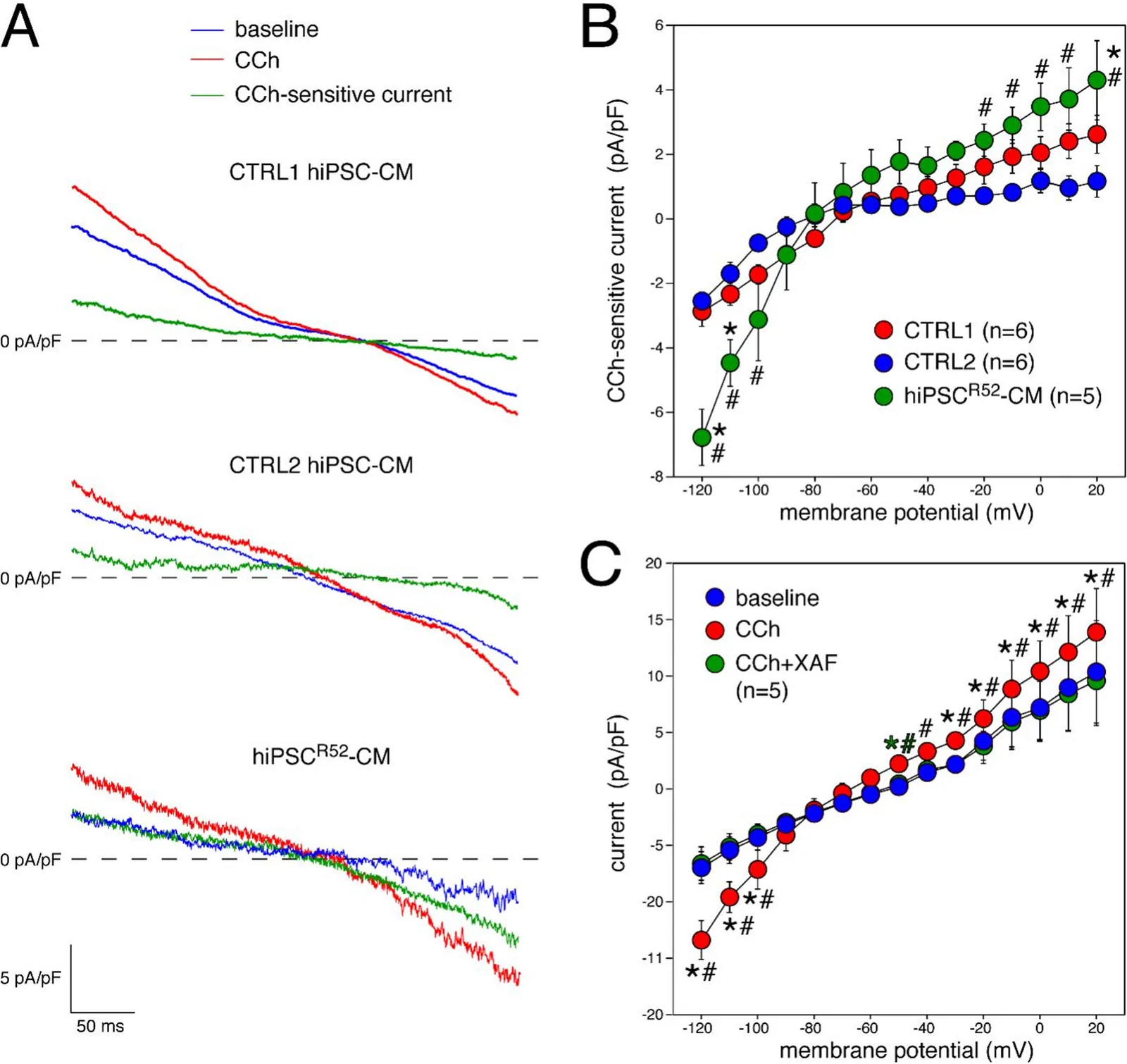
Effect of CCh on RA-treated hiPSC-CMs**. A** Typical currents of all three hiPSC-CMs lines during a RAMP protocol (top panel) in absence (baseline) and presence of 10 µM CCh, as well as the CCh-sensitive currents. **B** Average I-V relationships of the CCh-sensitive current. * *P* < 0.05 CTRL1 vs. hiPSC^R52L^-CMs; ^#^ *P* < 0.05 CTRL2 vs. hiPSC^R52L^-CMs (Two-way repeated measures (RM) ANOVA followed by Bonferroni post-hoc test). **C** Average I-V relationships of hiPSC^R52L^-CMs under baseline, in presence of CCh and in the presence of CCh and XAF-1407. XAF-1407 reversed the CCh effects. * *P* < 0.05 CCh vs. baseline; ^#^ *P* < 0.05 CCh vs. CCh + XAF-1407 (Two-way repeated measures (RM) ANOVA followed by Bonferroni post-hoc test)

Next, we tested I_K, ACh_ densities upon GIRK stimulation in our three RA-treated hiPSC-CM lines. To this end, we measured membrane currents in absence and in the presence of 10 µM CCh during our descending ramp protocol. Figure 3 shows typical examples in CTRL1-, CTRL2-, and hiPSC^R52L^-CMs. CCh activated a membrane current in all three hiPSC-CMs line (Fig. 3, A and B). However, the CCh-sensitive current was significantly larger in hiPSC^R52L^-CMs as compared to both CTRL hiPSC-CM lines (Fig. 3B). In hiPSC^R52L^-CMs, we tested whether the CCh effects could be reversed by I_K, ACh_ blockade. Figure 3C shows the I-V relationships under baseline, in the presence of CCh, and in the presence of CCh with XAF-1407. Application of 100 nM XAF-1407 reversed the effects of CCh. Thus, hiPSC derived cardiomyocytes with the R52L *GNB2* variant have a larger I_K, ACh_ density.

### GIRK channel stimulation results in more pronounced AP changes in RA-treated hiPSC^R52L^-CMs than in control hiPSC-CMs

**Fig. 4:**
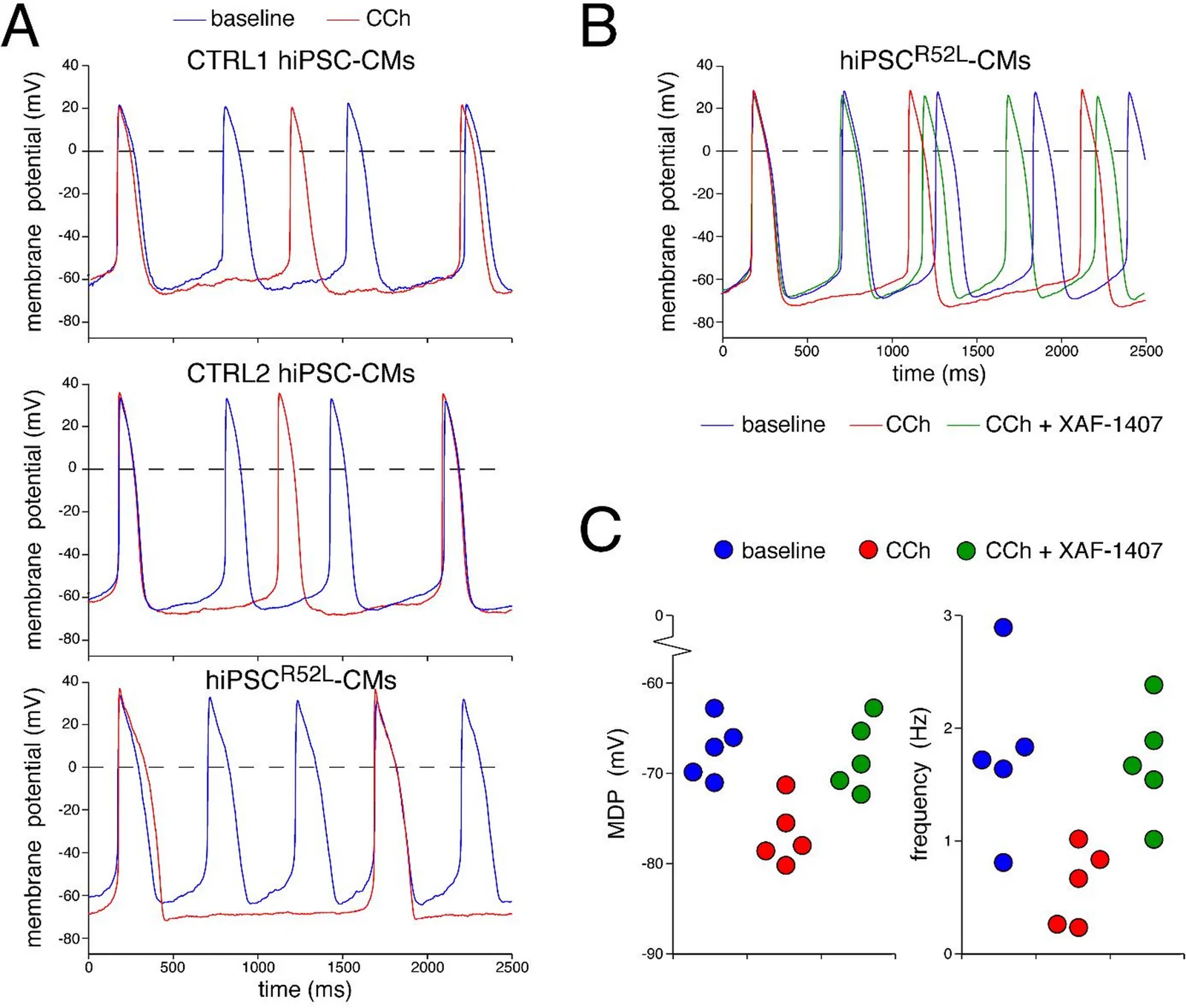
Effect of CCh on RA-treated hiPSC-CMs.** A** Typical APs of all three hiPSC-CMs lines in absence (baseline) and presence of 10 µM CCh. **B** Typical APs recorded in a RA-treated hiPSC^R52L^-CM in absence (baseline), presence of CCh, and in presence of CCh + XAF-1407. **C** Dot plots of MDP and spontaneous frequencies in RA-treated hiPSC^R52L^-CMs under baseline, in presence of CCh, and in the presence of CCh + XAF-1407. XAF-1407 reversed the CCh effects

In a final series of experiments, we tested the functional consequences on the differences in I_K, ACh_ density upon GIRK stimulation. Therefore, we measured APs of RA-treated hiPSC-CMs in absence and presence of 10 µM CCh. CCh induced a statistically significant hyperpolarization of the MDP and decrease in spontaneous pacing frequency in all hiPSC-CMs lines, as can be inferred from the typical examples in Fig. 4 and the summarized data of Table 3. In addition, V_max_ was increased in response of CCh, due to the larger sodium current availability in response of the MDP hyperpolarization [12]. Interestingly, the CCh-induced MDP hyperpolarization was more pronounced in hiPSC^R52L^-CM compared to CTRL hiPSC-CMs lines (8.7 ±1.7 (hiPSC^R52L^-CM), 2.2 ±0.6 (CTRL1), and 2.1 ±0.8 (CTRL2) mV (Kruskal-Wallis followed by Dunn’s test)), resulting in significantly lower MDP and pacing frequency in hiPSC^R52L^-CMs in the presence of CCh (Table 3). In five hiPSC^R52L^-CMs, we tested the effects of XAF-1407 in the continued presence of CCh. XAF-1407 reversed the effects of CCh on AP parameters in hiPSC^R52L^-CMs (Fig. 4, B and C), indicating that it may be a useful tool in treatment of SND patients carrying the R52L *GNB2* variant. Thus, hiPSC^R52L^-CM have a more pronounced bradycardia phenotype (beating rate) upon GIRK stimulation, which could be pharmacologically rescued with I_K, ACh_ blockers.

**Table 3:** AP parameters of RA-treated hiPSC-CMs in absence and presence of 10 µM CCh

	CTRL1 hiPSC –CMs (*n* = 8)		CTRL2 hiPSC-CMs (*n* = 13)		hiPSC^R52L^-CMs (*n* = 6)	
	baseline	CCh	baseline	CCh	baseline	CCh
MDP (mV)	-68.8 ±0.8	-71.0 ±0.6*^,#^	-68.8 ±1.1	-70.9 ±1.1*^,#^	-67.5 ±1.3	-76.0 ±1.6*
APA (mV)	93.8 ±2.1	98.7 ±2.8*	94.6 ±9.8	96.3 ±3.7	87.0 ±5.7	100.3 ±7.3
AP Plat (mV)	69.8 ±8.1	75.9 ±7.4*	87.3 ±3.5	86.6 ±4.4	64.1 ±15.3	64.5 ±16.2
V_max_ (V/s)	59.3 ±19.3	88.1 ±23*	42.4 ±9.8	61.9 ±14.6*	24.5 ±7.1	84.3 ±22.6*
APD_20_ (ms)	25.6 ±8.4	25.9 ±7.5	48.0 ±5.1	45.0 ±6.3	50.0 ±20.3	34.6 ±19.0
APD_50_ (ms)	48.1 ±13.5	49.1 ±11.7	85.7 ±8.2	84.1 ±10.1	85.3 ±30.8	69.4 ±37.6
APD_90_ (ms)	115.4±25.6	88.2±14.5	132.3±12.1	133.3±15.0	190.6±29.0	153.9 ±34.7
Freq (Hz)	2.1±0.3	1.1±0.1*^,#^	1.9±0.2	1.1±0.1*^,#^	1.8±0.3	0.7 ±0.2*

Average ±SEM. MDP = maximal diastolic potential; AP, action potential; APA = AP amplitude; AP plat = plateau amplitude measured at 20 ms after V_max_; V_max_ = maximal AP upstroke velocity; APD_20_, APD_50_, APD_90_ = AP duration at 20, 50, and 90% of repolarization; Freq = frequency. * *P* < 0.05 baseline vs. CCh (Mann-Whitney test); ^#^ *P* < 0.05 vs. hiPSC^R52L^-CMs (Kruskal-Wallis followed by Dunn’s test)

## Discussion

### Overview

In this study, the implications of the p.Arg52Leu *GNB2* gene variant were characterized in a patient-derived disease model using differentiated hiPSC-CMs of a patient carrying the *GNB2* variant and suffering from SND [14, 22]. Furthermore, we compared the electrophysiological properties to those of two unrelated healthy control hiPSC-CM cell lines (CTRL). RA application during the differentiation was used to generate hiPSC-CMs with robust expression of I_K, ACh_ (Fig. 3), which makes them useful for studying GIRK-related channelopathies. Neither CTRL, nor mutant hiPSC^R52L^-CMs, have a constitutively active I_K, ACh_ without GIRK stimulation (Fig. 2; Table 2). However, upon GIRK stimulation with CCh, the I_K, ACh_ density in hiPSC^R52L^-CMs was significantly larger as compared to CTRL hiPSC-CMs (Fig. 3). This resulted in more pronounced effects of CCh on APs, resulting in a more hyperpolarized MDP and a slower spontaneous pacing frequency in the presence of GIRK stimulation (Fig. 4; Table 3). In addition, blockade of I_K, ACh_ with XAF-1407 was capable to restore the GIRK gain-of-function of the hiPSC^R52L^-CMs (Fig. 4) and thereby paved the way for a specific, pharmacologic treatment.

### GIRK stimulation results in larger I_K, ACh_ in hiPSC^R52L^-CMs than in controls

In absence of GIRK stimulation, the AP properties of hiPSC^R52L^-CMs were largely similar to two unrelated healthy (CTRL) hiPSC-CMs lines (Table 1). In addition, baseline I_K, ACh_ activity was neither observed in the CTRL hiPSC-CMs nor in hiPSC^R52L^-CMs (Fig. 2; Table 2), thus the *GNB2* p.Arg52Leu variant itself did not trigger basal I_K, ACh_ channel activation. However, GIRK stimulation with 10 µM CCh, resulted in a larger I_K, ACh_ density in hiPSC^R52L^-CMs as compared to the CTRL hiPSC-CMs (Fig. 3), indicating that the R52L *GNB2* variant resulted in a I_K, ACh_ gain-of function during GIRK channel stimulation. This is in line with previous findings in heterologous cell expression systems [14], although here an increase in I_K, ACh_ was already observed at baseline, thus without GIRK stimulation. Likely, this is related to differences between the patient-derived hiPSC-CMs used here and heterologous, non-patient-derived cellular expression systems, such as HEK cells and oocytes. In our RA-treated hiPSC-CMs, there is no basal I_K, ACh_ present when the cells are not stimulated with CCh, in agreement with previous observations in stem cells [13, 16, 26]. These findings in stem cells is similar to atrial CMs harvested from patients with chronic atrial fibrillation (cAF) which also exhibited no effects on basal I_K, ACh_, when treated with pertussis toxin (PTX) to inactivate G_i_-proteins [39]. In contrast, Stallmeyer and colleagues [14] recorded already Kir3.1/Kir3.4 currents under their ‘baseline’ conditions in heterologous expression systems, which is also found by others [40]. Co-transfection with wild-type *GNB2* increased this basal-active Kir3.1/Kir3.4 current and this was even more pronounced by the R52L *GNB2* variant. While Stallmeyer et al. suggested that the R52L *GNB2* variant resulted in a sustained activation of GIRK channels, it is in fact an increase of already open Kir3.1/Kir3.4 current, similar as in our conditions with GIRK stimulation in our CM environment. Furthermore, the R52L *GNB2* variant has large similarities with the previously reported Ser81Leu variant in the *GNB5* gene causing an autosomal-recessive syndrome with SND and cognitive disability [40]. Upon application of CCh to stimulate I_K, ACh_, the S81L *GNB5* variant was electrophysiologically analyzed in hiPSC-CM in controls and in heterozygous and homozygous state.

While AP parameters in the absence of CCh did not differ between all three hiPSC lines, the cellular hyperpolarization, AP shortening at 20% duration as well as slowing of spontaneous beating rate were all more pronounced in S81L^homo^ compared to heterozygous and WT cell lines [13]. In particular, a reduction of the spontaneous beating rate was also measured in S81L^het^ after CCh administration, however to lower extent, indicating a pathological effect in a gene dosage dependent manner for GNB5. Anticipate a similar electrophysiological and phenotypic effect for the R52L variant in *GNB2* in homozygous state remains to be elucidated.

### GIRK stimulation results in a severe bradycardic phenotype in hiPSC^R52L^-CMs

Our hiPSC-CMs model is suitable to study the implications of the enhanced I_K, ACh_ on spontaneous pacemaker formation. We found a more pronounced effect of CCh on MDP and spontaneous pacing frequency, resulting in a severe bradycardic phenotype in hiPSC^R52L^-CMs (Fig. 4; Table 3). This is largely similar as found by Veerman and colleagues [13] were a *GNB5* gain-of-function variant resulted also in a severe impaired pacemaker generation, as outlined above. While we performed studies in singel cells and observed impaired pacemaker generation, impaired impulse conduction between the SAN and atria may also contribute to the SND of the patients. At least, computer simulation have demonstrated that an increased I_K, ACh_ reduces the excitability of atrial cells [13], thereby further contributing to the bradycardic phenotype in patients. While we found a gain-of function with the *GNB2* variant, *Gnb2* knock-out in mice showed the opposite with increased heart rate [41].

We cannot exclude that the SND in patients is partially due to neuronal defects, such as in the right stellate ganglion, which modulate heart rate significantly. *Gnb2* is also present in neurons [20], and similar with *GNB5* mutations [42], several *de novo GNB2* missense variants are associated with intellectual disability [42, 43]. For example, a comprehensive case study recently report the occurrence of five *de novo* missense *GNB2* variants in 12 unrelated individuals suffering from developmental delay and intellectual disability with variable dysmorphism and extraneurologic features [43]. All detected variants were located at the universally conserved shared interface with the Gα subunit. This finding led to the assumption that the molecular interaction between the G protein and its binding partners was impaired causing the observed phenotype. Thus, this case study demonstrated the possibility that variants in *GNB2* can cause a multisystemic phenotype associated by neurodevelopmental disorders with variable syndromic features. Unfortunately, we do not have enough clinical data from the affected family harboring the R52L GNB2 variant to confirm this hypothesis [43]. Further studies are needed with animal models, or neuronal iPSCs, to validate the hypothesis that R52L *GNB2* variant may also affect neuronal heart rate control.

### Clinical implications

Permanent pacemaker implantation is the first-line treatment for symptomatic patients with confirmed SND, accounting for 50% of pacemakers implanted in the United State (for review, see Hawks et al., 2021, and primary references cited therein [18]). By unravelling pathogenic mechanisms for SND and addressing these, e.g. by I_K, ACh_ channel blockade, this may pave the way for future, alternative clinical treatment options (Fig. 4). Selective blockers of I_K, ACh_ channels were also suggested as pharmacological treatment in other studies with GIRK gain-of-function mutations [13, 16] and in mice with SND [44]. Currently, various selective I_K, ACh_ channel blockers have been developed [45–49], but to our knowledge, not yet tested on patients with SND in clinical trials. Further studies are needed to address this issue.

### Limitations

We have used hiPSCs from a patient with the R52L GNB2 variant, and unrelated healthy hiPSCs (CTRL1 and CTRL2) served as control hiPSC lines. Ideally, the patient line should be corrected, which in the present study was done, but unfortunately did not resulted in successful electrophysiological experiments due to differentiation issues or halted patch clamp experiments due to karyotype defects. Inherent genetic background differences across cell lines might confound the observed cellular phenotypes and therefore some caution is needed for drawing firm conclusions about the GNB2 variant. We observed a larger IK, ACh activity in the GNB2 hiPSC-CMs, but mRNA and protein expression of Kir channels was not studied. A higher expression of Kir channels in the GNB2 variant line can therefore contribute to the increased GIRK activation although in our in heterologous cell expression system experiments with fixed amount of Kir channels [14] the increased activity was only attributed to the GNB2 variant. Furthermore, the sample sizes was at the lower end in some experiments, although it were paired experiments with drugs. In addition, we used hiPSC-CMs as a surrogate for pacemaker cells, similar to the studies employed by Veerman et al. [2019], Giannetti et al., [2021], and Kayser et al. [2023] [13, 16, 25]. The hiPSC-CMs were differentiated with RA, but the development and use of hiPSC-CM models is still fast evolving and currently, some differentiation protocols have been published to create SAN-like hiPSC-CMs [31, 50, 51]. Unfortunately, these protocols were not available at the start of our study. Nevertheless, the effects of CCh on spontaneously activity in SAN-like and atrial-like hiPSC-CMs are similar [31], but the recently developed SAN-like hiPSC-CMs model may facilitate in vivo pacemaking-related studies in future. The clinical significance of the presented results is limited by the fact that this study relies exclusively on single-cell in vitro data and should therefore be interpreted cautiously.

### Conclusions

In a CM environment, the R52L *GNB2* variant results after GIRK stimulation in a larger I_K, ACh_ density resulting in a more pronounced bradycardic phenotype, which could be treated with I_K, ACh_ blockers indicating a potential therapeutic implication in patients suffering from GIRK channel related severe bradycardia.

## Data Availability

The datasets generated and/or analyzed during the current study are available from the corresponding author on reasonable request.
